# Nonaqueous Synthesis of Low-Vacancy Chromium Hexacyanochromate

**DOI:** 10.1021/acs.inorgchem.4c03856

**Published:** 2024-11-18

**Authors:** Maximilian Schart, Ramón Torres-Cavanillas, Samuel Wheeler, Kevin Hurlbutt, Pascal Manuel, Dmitry Khalyavin, Ruomu Zhang, David Vincent, Xavier Rocquefelte, George Volonakis, Andrew Goodwin, Lapo Bogani, Mauro Pasta

**Affiliations:** † Department of Materials, University of Oxford, Oxford OX1 3PH, U.K.; ‡ ISIS Pulsed Neutron and Muon Source, STFC Rutherford Appleton Laboratory, Harwell Campus, Didcot, Oxon OX11 0QX, U.K.; § Univ Rennes, ENSCR, INSA Rennes, CNRS, ISCR (Institut des Sciences Chimiques de Rennes), UMR 6226, Rennes F-35000, France; ∥ Inorganic Chemistry Laboratory, Department of Chemistry, University of Oxford, Oxford OX13QR, U.K.

## Abstract

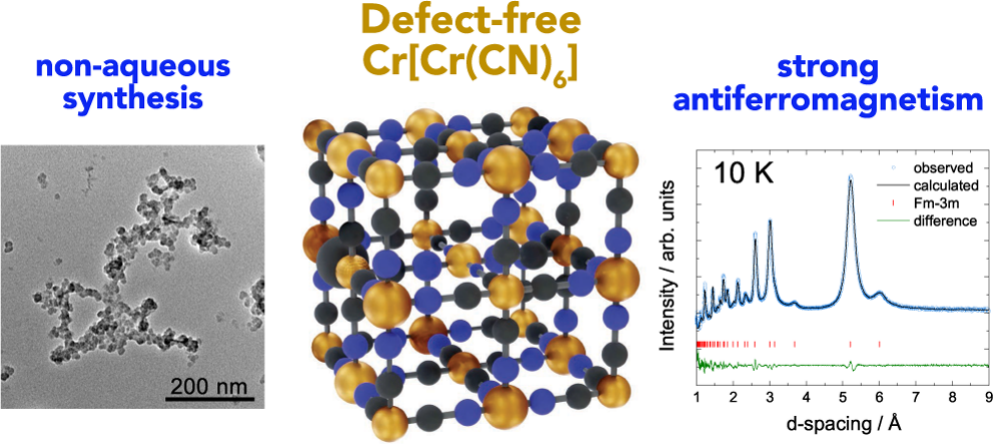

Prussian blue analogues (PBAs) are a highly tunable family of materials with properties suitable for a wide variety of applications. Although their straightforward aqueous synthesis allows for the facile preparation of a diverse set of compositions, the use of water as the solvent has hindered the preparation of specific compositions with highly sought-after properties. A typical example is Cr[Cr(CN)_6_]: its predicted strong magnetic interactions have motivated many attempts at its synthesis but with limited success. The lack of control over vacancies, crystallinity, and the oxidation state has prevented the experimental validation of its theoretical magnetic properties. Here, we report the nonaqueous synthesis of vacancy-suppressed, nanocrystalline chromium hexacyanochromate. The control over vacancies and the oxidation state leads to stronger magnetic interactions with a markedly increased absolute Weiss temperature (Θ = −836(6) K) and magnetic ordering temperature of (240 ± 10) K. Our results challenge the notion of the solvent as merely reaction medium and introduce a pathway for exploring moisture- and air-sensitive PBA compositions.

## Abstract 2

Here, we report the nonaqueous synthesis of nanoparticles of the elusive Cr[Cr(CN)_6_] Prussian blue analogue. By using our protocol, a less defective Prussian blue analogue structure is achieved, resulting in an enhanced antiferromagnetic response.

## Introduction

The discovery of high magnetic ordering temperatures in PBAs about 30 years ago has caused a surge of interest in their use in magnetic devices. Since then, a number of magnetic phenomena have been found in these materials, ranging from different spin arrangements, multiple compensation temperatures, light- and moisture-induced magnetism^1−3^ and – arguably in the center of interest–above-room temperature magnetic ordering.^4^ The vast spectrum of observed magnetic properties is enabled by the material system’s compositional variability. PBAs have a general formula of A_*x*_P*^i^*[R*^j^*(CN)_6_]_1–*y*_ · *w* Sol (abbreviated as A_*x*_P*^i^*[R*^j^*]), where the choice of alkali metal cation A, transition metal ions in octahedral coordination to the nitrogen and carbon atom of the bridging cyanide ligands, P and R and their respective oxidation states *i* and *j* as well as the fraction of hexacyanometallate anion vacancies in the structure *y* open up a wide composition space. Residual solvent (Sol) from synthesis can be present both within zeolitic pores and bound to under-coordinated P-site transition metal species surrounding vacancies.

The theoretical framework describing the effect of varying compositions on magnetic properties was presented by Néel,^5^ condensed in the following relation for the magnetic critical temperature (*T*_C_):
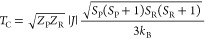
1where *Z*_P_, *Z*_R_ are the number of magnetic neighbors of the P and R transition metals, *J* is the exchange interaction parameter, *S*_P_ and *S*_R_ are the respective spins and *k*_B_ is the Boltzmann constant.^6^*Z* is directly related to the PBA’s vacancy fraction *y* via *Z* = 6–6*y*, indicating that a higher number of vacancies depresses *T*_C_. *J* and *S*_P/R_ are determined by the choice of spin centers P and R. In PBAs, the spin centers’ octahedral ligand environment leads to antiferromagnetic interactions (*J* < 0) between unpaired electrons in neighboring t_2g_ orbitals and ferromagnetic interactions (*J* > 0) between neighboring t_2g_ and e_g_ orbitals, as described by Kahn and Hoffmann.^7−9^

These theories have demonstrated excellent agreement with observations. The highest ferromagnetic ordering temperature found in PBAs is 90 K in the material CsNi[Cr],^10^ which possesses full occupation and solely t_2g_–e_g_ interactions. This led to a decades-long race for optimized materials with t_2g_^3^–t_2g_^3^ electronic configuration, for which the strongest antiferromagnetic coupling is predicted.^6^ The efforts culminated in the discovery of near-ideal KV^*II*^[Cr^*III*^], a t_2g_^3^–t_2g_^3^ PBA which exhibits ferrimagnetism with a *T*_C_ of 376 K.^4^ More recently, novel coordination compounds have been proposed with higher ordering temperatures, including CrCl_2_(pyz)_2_, which exhibits a *T*_C_ of 515 K.^11−15^ Unfortunately, all the room-temperature molecular magnets known to date are highly air-sensitive.

A possible candidate is Cr^*III*^[Cr^*III*^], a highly sought-after PBA composition previously targeted for its t_2g_^3^–t_2g_^3^ electronic configuration and stability in air and moisture. Previous synthesis attempts have resulted in highly vacant or amorphous materials displaying properties different to those predicted for a vacancy-free, crystalline material.^16−22^

In this study, we introduce a nonaqueous chemical synthesis strategy to prepare chromium hexacyanochromate in its fully oxidized state. Physicochemical characterization confirms that the material contains negligible hexacyanochromate vacancies. This approach allowed us to explore the magnetic properties of crystalline Cr^*III*^[Cr^*III*^] in depth via a combination of neutron powder diffraction and magnetic measurements. We obtain a *T*_C_ of (240 ± 10) K for the material, which is in good agreement with chromium hexacyanochromates PBAs. We show that vacancy minimization leads to the highest absolute Weiss temperature reported for PBAs, with Θ = (−836 ± 6) K. Our experimental results are supported by first-principles calculations based on density functional theory (DFT).

## Results and Discussion

### Synthesis and Characterization

PBAs are known for their straightforward precipitation synthesis from aqueous solutions. However, the chemistry of the precursors for synthesizing chromium hexacyanochromate demands a different approach. First, solvated Cr^3+^ is not labile enough to incorporate into the PBA framework, effectively blocking the direct formation of the oxidized Cr^*III*^[Cr^*III*^]. Second, the reduced Cr(CN)_6_^4–^ reaction intermediate is unstable against ligand dissociation in aqueous solution.^23,24^ Third, the oxidation potentials of neither the divalent chromium precursors nor the formed material are contained within the electrochemical stability window of water.^25^ To overcome the first limitation, Cr^*III*^[Cr^*III*^] can be obtained via the synthesis of the reduced material followed by subsequent oxidation, while the second and third limitations are directly related to the use of an aqueous medium and can therefore be addressed by selecting a more suitable nonaqueous solvent. Candidates must be electrochemically inert and highly polar in order to form stable solutions of divalent chromium precursors and to stabilize the Cr(CN)_6_^4–^ complex.^26^

The organic solvents formamide (FA) and *N*-methylformamide (NMF) meet these requirements, exhibiting higher dielectric constants (ε_FA_ = 109.6 and ε_NMF_ = 182.4)^27^ and extended stability against reduction compared to water. The synthesis of reduced A_2_Cr^*II*^[Cr^*II*^] (A = Na, K, Rb) is attempted from Cr^2+^ and simple cyanide in both of these solvents, according to the two-step reaction:^33^
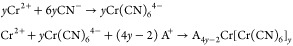
2where (1–*y*) is the fraction of hexacyanochromate vacancies in the material. The reduced PBA crystallizes in both FA and NMF solvents (Figure S1). However, the formed materials exhibit structural differences. In FA, materials with high vacancy content form (discussion in Supporting information (SI)). In NMF, such vacancies are suppressed for A = Rb. Syntheses in the same medium using smaller counterions (A = Na, K) yield amorphous precipitates.

Clearly, the nature of the solvent steers the reaction pathway and influences the product composition. The observations can be explained by consideration of enthalpic contributions to the material formation. Vacancies are regions in the PBA framework where under-coordinated transition metal cations face and repel each other. Coordinated solvent molecules provide electrostatic shielding, thus easing the associated enthalpic toll. If the solvent’s molecular size becomes too large, as observed with NMF, steric hindrance within vacancies prevents this shielding effect. Consequently, vacancy formation becomes enthalpically unfavorable and is thereby inhibited. This argument suggests that vacancy suppression in NMF is directly caused by the solvent’s molecular size.

**Figure 1:**
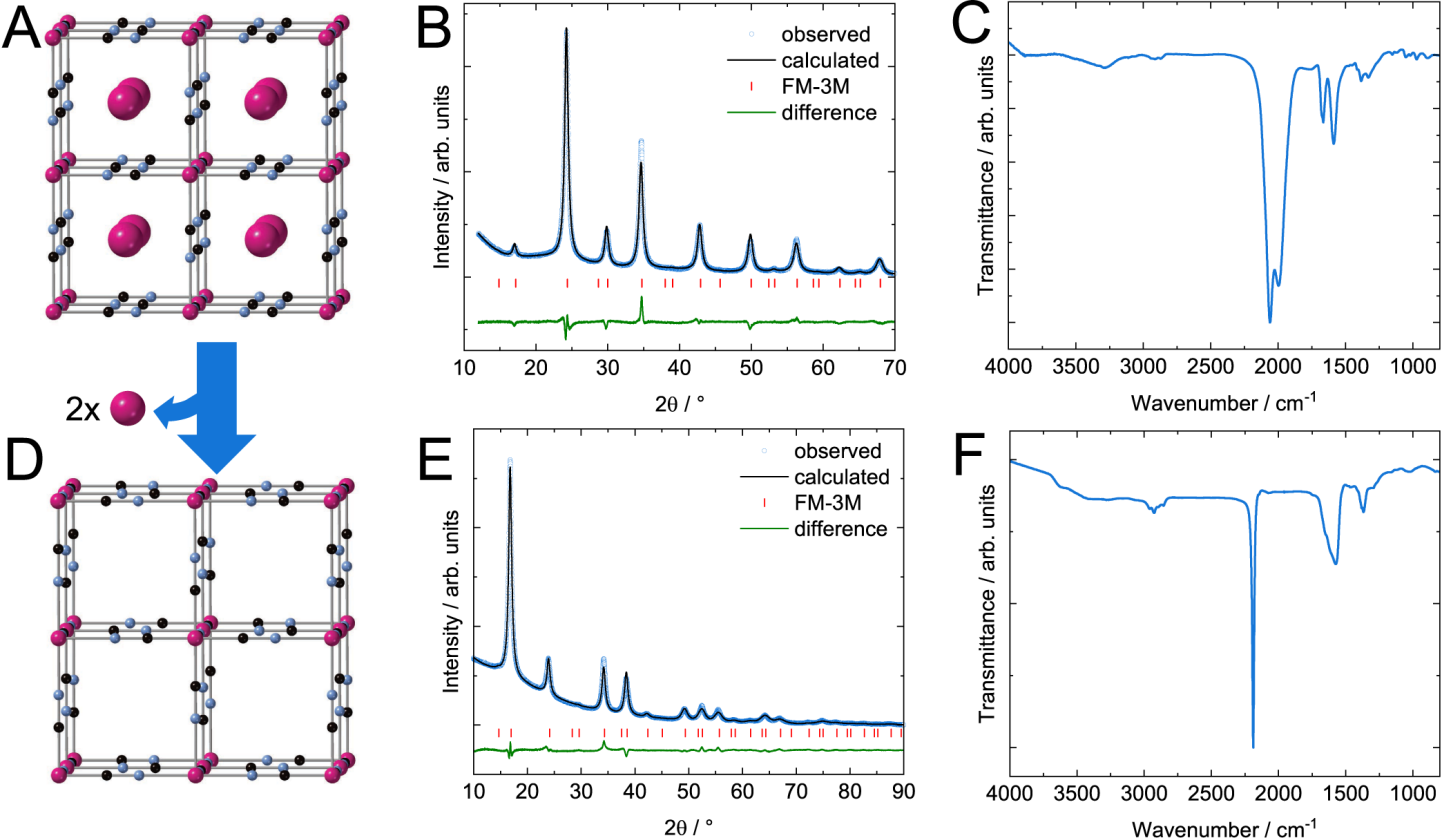
Physicochemical materials characterization. Model structure (A), PXRD (B) and IR transmission spectrum (C) of **(1)**. Model structure (D), PXRD (E) and IR transmission spectrum (F) of **(2)**. Downward facing arrow illustrates chemical oxidation under extraction of Rb^+^. The diffractograms of both materials can be indexed in the *Fm*3̅*M* space group. The oxidation state determines the amount of A^+^ ions inside the structure. Cyanide stretch frequencies are sensitive to the materials’ oxidation states. Higher oxidation states cause shifts of the ν(CN) band toward higher wavenumbers, from 2060 and 1997 cm^–1^ for **(1)** to 2186 cm^–1^ for **(2)**.

The reduced material **(1)** synthesized in NMF with Rb^+^ counterions is a black, nanocrystalline powder with particle sizes of 10 to 30 nm (Figures S2A and S3). A cubic crystal structure (*Fm*3̅*m*) with a lattice parameter of 10.31 Å is determined via powder X-ray diffraction (PXRD) (Figure 1A). Due to the almost equal X-ray scattering cross sections, disorder in the positions of C and N, resulting in a *Pm*3̅*m* space group and a lattice constant of 5.16 Å, would lead to indistinguishable XRD observations and can therefore not be ruled out. Ligand field effects, however, lead to the observation of ligand order in transition metal cyanide compounds and is therefore assumed. Rietveld refinement of the diffractogram and elemental analysis via inductively coupled plasma mass spectrometry (ICP-MS, see Figure S4) determine a composition of Rb_1.474(26)_Cr[Cr(CN)_6_]_0.997(19)_ (full set of refined parameters in Figure S6). Partial oxidation accounts for the deviation of the Rb:Cr ratio from the expected unity. This is supported by the presence of two distinct peaks at 2060 and 1997 cm^–1^ in the ν(CN) region (1900 to 2300 cm^–1^) of the material’s infrared spectrum (Figure 1C). Additional absorption peaks in the FTIR spectrum at <1700 and 2800 to 3000 cm^–1^ indicate the presence of residual NMF solvent.

In the second step, **(1)** is chemically oxidized to achieve the desired Cr^*III*^[Cr^*III*^]. We choose water as the oxidant because it ensures a fast interfacial Rb^+^ ion transfer into the solvated state and facilitates ionic transport within the PBA structure.^28^ The process follows the idealized reaction:
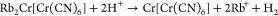
3This reaction consumes H^+^, which increases the pH value during oxidation. Since PBAs are unstable in basic pH, a dilute acidic solution is chosen for this step to provide an excess of protons.

**Figure 2:**
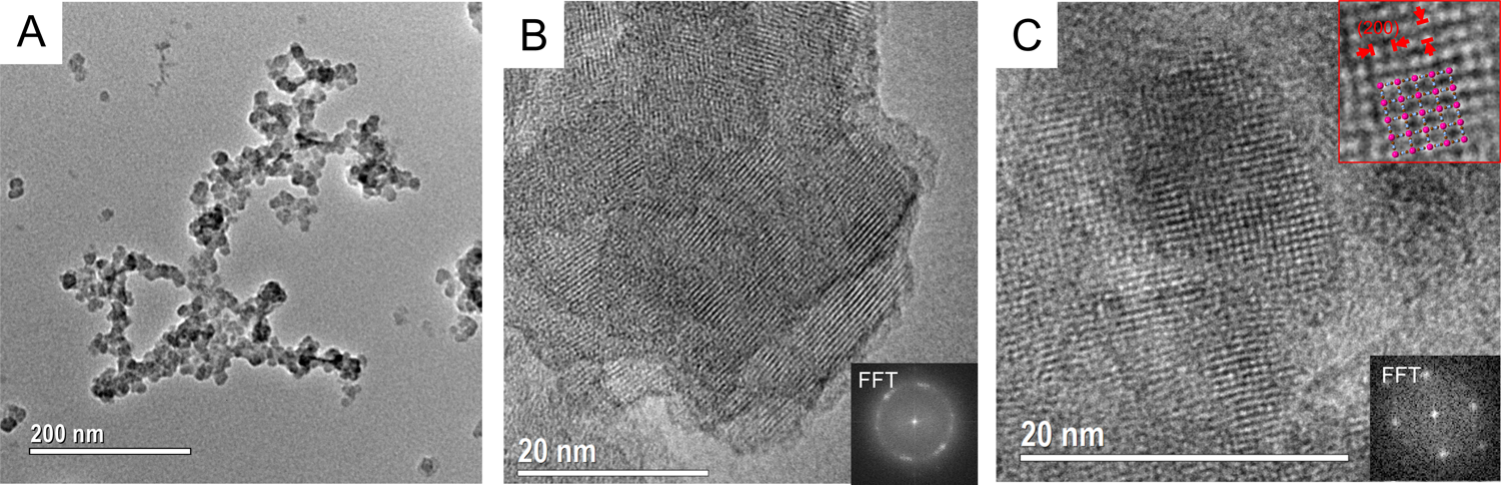
Transmission electron micrographs of **(2)**. Low-magnification imaging shows particle sizes of 10 to 30 nm (A). Continuous lattice fringes over ≈30 nm indicate the particles’ single-crystalline nature (B). The material’s cubic crystal structure is directly evident via imaging in [100] zone axis (C).

The oxidation yields a light brown, nanocrystalline powder material **(2)** (Figure S2B). PXRD shows that the material has a face-centered cubic crystal structure (*Fm*3̅*m*), with a lattice constant of 10.42 Å (Figure 1E). Again, when accounting for the possibility of ligand disorder, the observed diffractogram can be indexed in *Pm*3̅*m* space group, with a lattice constant 5.21 Å as discussed above. Elemental analysis via ICP-MS confirms that the Rb is entirely extracted upon oxidation, supporting the Cr full oxidation (table S4). Additionally, we explored the Cr oxidation state in compound **(2)** by measuring the Cr K-edge of the oxidized nanoparticles and comparing it with the Cr^*II*^[Co^*III*^]_2/3_ PBA (Figure S5). We chose Cr^*II*^[Co^*III*^]_2/3_ as the reference instead of **(1)** due to the ease with which **(1)** oxidizes during sample preparation. Remarkably, a shift of about 1 eV to lower energies, around 6008 eV, is observed in compound **(2)**, compared to our reference, which is consistent with the presence of Cr in the 3+ state.^29,30^ The spectra’s shape also aligns with the presence of Cr^III^.^31^ However, it should be noted that the presence of Cr^II^ impurities cannot be completely ruled out. Rietveld analysis of PXRD data quantifies a composition of Cr[Cr(CN)_6_]_0.996(7)_, a virtually defect-free Cr^*III*^[Cr^*III*^] (full set of refined parameters in Figure S7). This composition shows that no vacancies are introduced upon oxidation. Transmission electron microscopy (TEM) images (Figure 2) show particles of about 20 nm in diameter (Figure S8). High-resolution micrographs show coherent lattice orientation over about the entire particle, evidencing that the particles are single-crystalline (Figure 2B). The material’s cubic crystal structure is directly visible in images along the [100] zone axis (Figure 2C) and confirmed by selected area electron diffraction (Figure S9).

The infrared spectrum (Figure 1F) of **(2)** shows a single, intense peak in the ν(CN) region at 2186 cm^–1^. The peak shift toward higher wavenumber suggests that the [Cr^II^(CN)_6_]^4–^ has been fully oxidized to [Cr^III^(CN)_6_]^3–^.^32,33^

**Figure 3:**
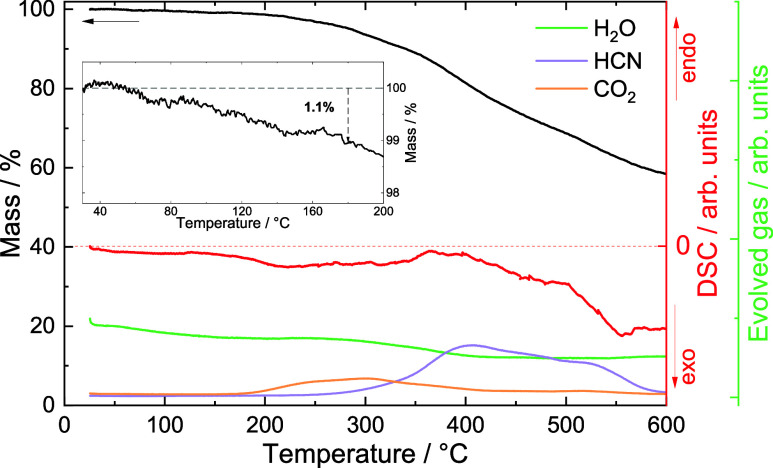
Thermal analysis of **(2)**. The material loses 1.1% of its mass likely due to solvent evaporation before a temperature of 180 °C. At this temperature, the DSC signal displays the onset of an exothermic process. The coupled gas analysis registers the evolution of CO_2_ at this temperature, which indicates material decomposition. Beyond 300 °C, the decomposition becomes more severe, with rapid sample mass decay and the typical evolution of HCN from the material. Throughout the measurement, the water signal remained roughly constant at background levels.

Additional peaks are due to residual NMF and water (broad ν(OH) peak at 3620 cm^–1^). Thermogravimetric analysis quantifies a mass loss of 1.1% before the material’s thermal decomposition at 180 °C, which can be ascribed to the remaining solvent (Figure 3). Such small quantities are characteristic of PBAs with low vacancy concentrations, as full occupation prevents available sites for solvent molecule coordination.^34^

### Magnetic Properties

Numerous studies have demonstrated the validity of simple orbital and mean field theories by Kahn, Hoffmann and Néel to predict magnetic ordering in PBAs.^5,7,9^ At their core are predictions of strong antiferromagnetic exchange interactions between neighboring t_2g_^3^–t_2g_^3^ spin centers, resulting in high ordering temperatures when magnetic coordination is maximized. Based on these established theories, the synthesized vacancy-less, oxidized Cr^*III*^[Cr^*III*^] should be an air-stable, high-temperature molecular magnet.

DFT calculations employing hybrid functionals, determine equal spins of *S* = 3/2 for both Cr_C_ and Cr_N_, (see the projected density of states in Figure S10). The spin alignment of the two interpenetrating sublattices is computed to be antiparallel to one another (Figure S11A), consistent with predictions from orbital theories and previous studies. The absence of vacancies and antiparallel alignment of equal spin carriers suggests antiferromagnetic order in **(2)** below the critical temperature *T*_C_. Due to spin compensation, experimentally observed magnetic moments are expected to be small or even zero in this system, making defects or impurities the main source of magnetic response. Consequently, investigating the magnetic properties of antiferromagnetic systems is extremely difficult and prone to misinterpretation.^35,36^ This is especially true for antiferromagnetic nanoparticles, for which even surface phenomena can overshadow the antiferromagnetic signal.^37−39^ To ensure correct interpretation, we complement magnetic measurements with powder neutron diffraction (PND).

**Figure 4:**
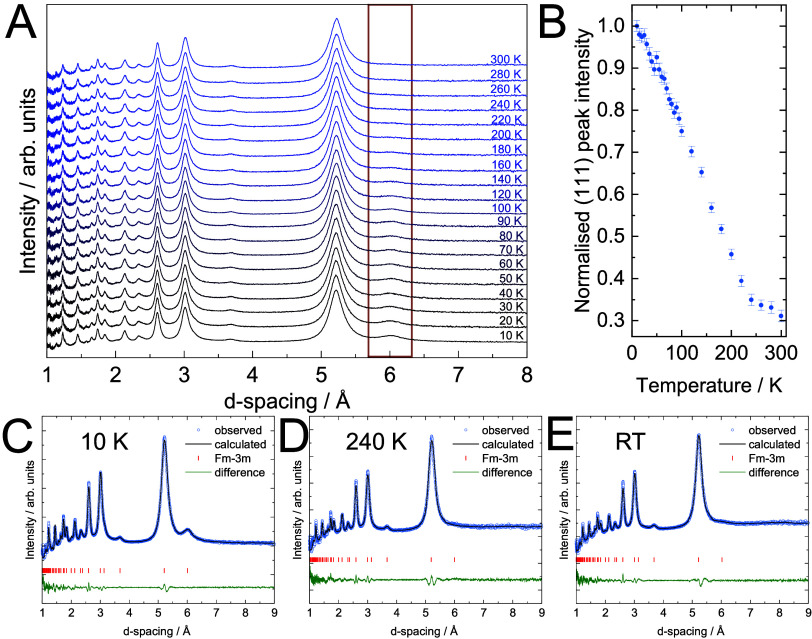
Neutron powder diffraction of **(2)**. Temperature-dependent neutron diffraction patterns measured at temperatures from 10 to 300 K (A). The dominant magnetic contribution to the diffraction pattern is observed at the (111) reflection position (6.0 Å, red box). Temperature-dependence of the (111) peak intensity extracted using Le Bail fitting tracks the evolution of long-range magnetic order(B). The magnetic contribution vanishes above (240 ± 10) K, marking the magnetic ordering temperature. Neutron diffraction patterns can be refined using magnetic Rietveld fits in the *Fm*3̅*m* space group throughout the measured temperature range (C–E).

Figure 4A shows the results of PND experiments measured over a temperature range from 10 to 300 K. The nuclear diffraction agrees with the model refined from PXRD. Superimposed, we obtain a significant temperature-dependent magnetic diffraction at the (111) reflection (*d* = 6.0 Å). Its presence identifies magnetic order in the material in the predicted spin arrangement (as discussed in Figure S11B). The temperature-dependence of the (111) peak intensity is shown in Figure 4B. Its intensity decreases with temperature and vanishes at (240 ± 10) K. This temperature marks the loss of magnetic order and thus the material’s *T*_C_. This value is in reasonable agreement with the *T*_C_ of 323 K predicted by employing classical Monte Carlo simulation and the ab initio calculated *J* (see Figure S12). The theoretical overestimation of ordering temperatures is quite common, particularly when dealing with nanoparticles, as simulations often fail to account for finite-size effects.^40^

No significant magnetic contribution is observed at smaller *d*-spacing because of the rapid decay of the magnetic form factor. Individual PND patterns at 10, 240 and 309 K are shown in Figure 4C–E. A magnetic moment of (2.78 ± 0.22) μ_B_ per Cr center is refined at 10 K, which is in excellent agreement with Cr^III^ spin centers in t_2g_^3^ configuration.^41,42^

**Figure 5:**
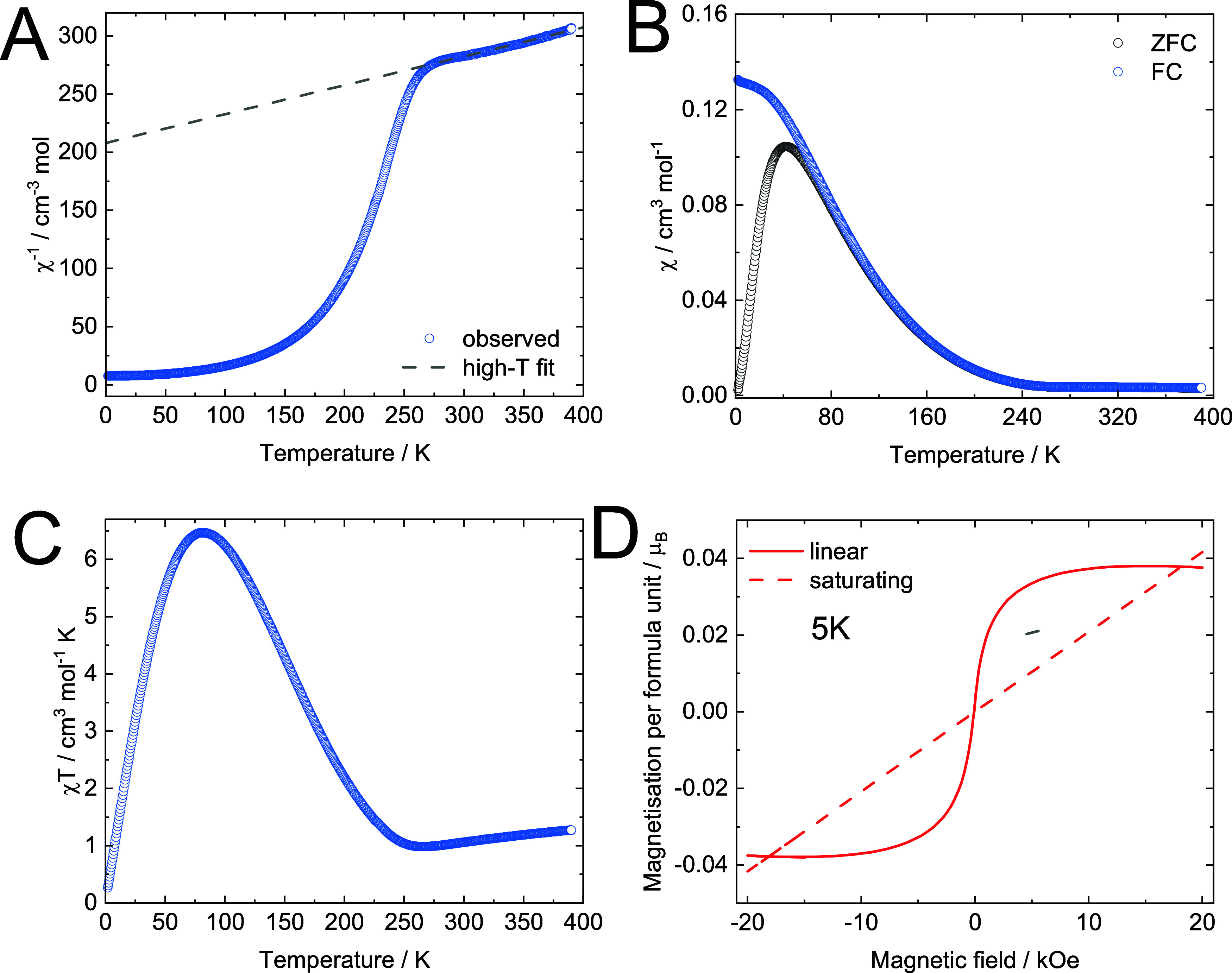
Temperature- and field-dependent magnetic measurements of **(2)**. Temperature-dependent susceptibility data represented as χ^–1^ vs T (A), χ vs T including results from zero field cooled (ZFC) and field cooled (FC) experiments (B) and χ*T* vs *T* (C). Field-dependent magnetization curve at 5 K deconstructed into linear and saturating parts (D). The increase of χ at 240 K toward lower temperatures is indicative of the onset of magnetic ordering with noncompensation. In the χ*T* vs *T* curve, a minimum is found at 265 K, revealing that the ordering is of antiferromagnetic type. In the high-temperature region above 270 K, the data is well described by the Curie–Weiss law, as shown in the χ^–1^ vs *T* curve. From saturation magnetization at 5 K, a noncompensation of 0.038 μ_B_ per formula unit is determined.

On the basis of the gained insights from PND, the magnetic properties of **(2)** are further studied via SQUID magnetometry. The material’s temperature-dependent susceptibility exhibits Curie–Weiss behavior in the paramagnetic region above 270 K (Figure 5A). At 270 to 390 K, we obtain a Curie constant C of (4.02 ± 0.02) cm^–3^ mol^–1^ K, which is in good agreement with the ideal spin-only value of 3.8 cm^–3^ mol^–1^ K expected for the Cr^*III*^[Cr^*III*^] PBA. The fit finds a highly negative Weiss constant of Θ = (−836 ± 6) K, which is close to our computational predictions from both the mean field expression (Θ_DFT_ = −752 K) and the Monte Carlo simulations (Θ_MC_ = −718 K in Figure S13), confirming the exceptionally strong short-range antiferromagnetic coupling. It should be noted that the Θ value is approximate. This is because Curie–Weiss fitting has limitations for systems with very high ordering temperatures. To accurately reach the paramagnetic region, temperatures much higher than those achievable by a conventional superconducting quantum interference device (SQUID) are required. Nevertheless, the observed Θ, despite its inaccuracy, can be used for comparison with other chromium-based PBAs since they all exhibit ordering temperatures above 200 K and undergo similar fittings.^16,17^ In this context, our Weiss temperature is considerably more negative than those reported in other studies, reflecting the maximization of antiferromagnetic interaction pathways and magnetic coordination achieved in our nanoparticles.^16,17,20^

The χ*T* vs *T* curve (Figure 5C) exhibits a minimum at 265 K, below which the susceptibility rapidly increases (Figure 5B), indicating the onset of uncompensated ferrimagnetic ordering.^16,43^ The presence of uncompensated spins constitutes a deviation from the expected antiferromagnetic behavior and indicates compositional nonideality. The field-dependent magnetization curves in this temperature region feature a weak hysteresis above 20 K, which vanishes completely above 200 K (Figure S14). This is in good agreement with the ferrimagnetic ordering of around 240 K extracted from quasi-static (DC) magnetization and neutron scattering.

The extent of noncompensation of **(2)** is studied from its low-temperature saturation magnetization *M*_sat_ (Figure 5D). At 5 K, we obtain a *M*_sat_ of 0.038 μ_B_ per formula unit after correcting for linear contributions, which could be caused by 1.3% Cr(CN)_6_^3–^ vacancies, 3.8% of partly reduced Cr^II^, surface effects as described by Néel^37^ or a mixture of them. The exact origin cannot be determined from the employed analysis. However, the small extent of nonideality attests to the negligible vacancy concentration in the material. Additional discussion and a full list of magnetic parameters are presented in the SI.

Notably, compared to the previously reported defective Cr^*II*^[Cr^*III*^]_2/3_ synthesized with the same protocol,^16^ sample **(2)** have a similar ordering temperature. However, the defective sample shows a much stronger magnetic response, as seen in Figure S15. This is because our Cr^*III*^[Cr^*III*^] system allows for more compensated antiferromagnetic coupling. However, the similar *T*_C_ values might be due to two main factors: (i) the presence of Cr(II) impurities, and (ii) uncompensated spins on the nanoparticle surfaces along with finite-size effects.^44^ Both factors may explain the small ferrimagnetic response observed using SQUID and PND.

## Conclusions

Our results present the use of organic reaction media to synthesize the so far inaccessible vacancy-free Cr^*III*^[Cr^*III*^] and show how the choice of solvent can influence vacancy formation in PBA materials. These insights have the potential to open up new avenues in PBA research. First, alterations of synthesis media can advance the synthesis of air- and moisture-sensitive PBA compositions, with which unanswered scientific questions can be resolved. Second, the shown solvent-mediated vacancy suppression can enhance the performance of PBAs in magnetic and energy storage applications. In the synthesized material, we measure a substantially increased absolute Weiss temperature, which underlines the importance of full occupation and crystallinity on magnetic interactions. The presented synthesis methodology encourages the use of nonaqueous media to investigate other water-sensitive PBA compositions and expand the range of this material family’s remarkable properties.

## Experimental Methods

### Chemicals

For the materials synthesis, CrCl_2_ (99.9%, anhydrous, Sigma-Aldrich), KCN (98%+, Sigma-Aldrich), RbCl (99%, Sigma-Aldrich) and *N*-methylformamide (99%, Sigma-Aldrich) were used. CrCl_2_ was used as purchased. KCN and RbCl were dried at 300 °C in vacuum (Büchi, Switzerland) prior to experiments. *N*-methylformamide (NMF) was dried using molecular sieves (3 Å, Sigma-Aldrich). All chemicals listed above were stored in an argon-filled glovebox (MBraun, Germany) after drying. For the chemical oxidation, nitric acid (ACS reagent grade, Sigma-Aldrich) and ultrapure water (18.2 MW cm, Merck Millipore) were used.

### Synthesis of (1)

The synthesis was carried out in an argon-filled glovebox. CrCl_2_ was dissolved in 12 mL of NMF to form a 200 mM solution. KCN and RbCl were dissolved together in 30 mL of NMF to form a 240 and 160 mM solutions, respectively. The two solutions were heated up to 180 °C on a hot plate and kept at this temperature during the reaction. Under vigorous stirring, the CrCl_2_ solution was added dropwise to the KCN and RbCl solution. After mixing, the reaction liquid was slowly cooled down to room temperature with continued stirring. The reaction liquid was sealed airtight and centrifuged to collect the precipitate of **(1)**. To remove unreacted precursors, **(1)** was washed two times with NMF and dried at 75 °C in a vacuum oven before characterization. Note that any precursor, neither **(1)**, were exposed to air at any stage of the synthesis or characterization. To produce the oxidized material **(2)**, it was further processed directly after washing with NMF without drying.

### Synthesis of (2)

A 0.1 M solution of HNO_3_ in ultrapure water was prepared. The synthesized material **(1)** was exposed to air, right before 60 mL of the dilute nitric acid solution was added to it. The dispersion was heated to 95 °C and stirred at this temperature for at least 2 days. Afterward, the material was collected by centrifugation, washed twice with ultrapure water, and dried at 140 °C under vacuum to produce **(2)**. The material was not allowed to rehydrate and was handled in the glovebox.

### X-ray Diffraction

Powder X-ray diffraction measurements were carried out using a Rigaku SmartLab diffractometer (Cu Kα source) for air-stable materials or using a Rigaku MiniFlex diffractometer operated in a N_2_-filled glovebox for air-sensitive materials. The data was analyzed using GSAS-II software for Rietveld refinement.^45^

### FTIR Spectroscopy

FTIR spectroscopy was performed using a Thermo Scientific Nicolet iS50 spectrometer operated in a N_2_-filled glovebox. Because of its high absorption, specimens of **(1)** were measured in transmission mode using KBr disks. The KBr was dried at 100 °C in vacuum and stored in an argon-filled glovebox prior to experiments. Specimen of **(2)** were measured in ATR mode. A background subtraction was performed for all measured data sets.

### Chemical Analysis

Elemental composition of the materials was measured using Inductively Coupled Plasma-Mass Spectrometry (ICP-MS) (Shimadzu ICPMS-2030). Specimens were prepared by dissolving small amounts of material in a 4:1 vol. mixture of concentrated HNO_3_ and H_2_SO_4_ in an Anton Paar Multiwave Go Plus microwave digester at 180 °C and diluted into a 2% HNO_3_ matrix.

### Electron Microscopy

Samples for electron microscopy were prepared by loading small amounts of dry material onto a carbon-coated Cu grid. TEM images were taken using a Jeol JEM-2100 microscope. SEM images were taken using a Zeiss Merlin SEM in transmission mode.

### Magnetic Properties Characterization

χ(*T*) was measured at 200 Oe using a Quantum Design MPMS-XL SQUID magnetometer equipped with a 7 T magnet and operating in the 2 to 390 K range. Diamagnetic contributions were corrected with the diamagnetic Pascal tables. *T*_C_ was calculated by linear extrapolation of *M*^2^(*T*) to *M* → 0, as suggested by molecular field theory.

### Neutron Diffraction

Powder neutron diffraction experiments in this work were carried out on the WISH beamline at the ISIS Neutron and Muon Source at the STFC Rutherford Appleton Laboratory in Oxfordshire, UK. The beamline is a time-of-flight (TOF) neutron diffractometer offering high resolution and a wide d-range. The sample (700 mg) was placed in a thin-walled vanadium can and mounted in a cryostat. Diffraction patterns were measured at 10 K intervals on cooling from 300 to 10 K. Multipattern Rietveld refinements were carried out using FullProf software. We proceeded to carry out Rietveld refinements of the full neutron powder diffraction data set, making use of the known crystal structure and assigning magnetic moments of equal size on both P- and R- sites, alternately polarized along the ± [100] directions. The corresponding ordered moment at base temperature was 2.78 ± 0.22 μB per Cr center. We allowed separate peak-shape parameters for nuclear and magnetic contributions to the diffraction pattern. In the analysis of the data presented in Figure 4A its normalization was done with respect to the highest peak. On the other hand, in Figure 4B, the (111) peak was independently fitted using a Gaussian function, with a background based on a constant value plus the tail of the Gaussian for the bright peak which is adjacent. The “Normalized (111) Peak Intensity” was determined by calculating the area under the (111) peak at each temperature and normalizing it by the peak area at 10 K, which corresponds to the highest observed intensity. Note that intensity at the (111) position is allowed even within the paramagnetic regime and arises from nuclear scattering contrast between the P-2 and R-sites of the PBA structure (e.g., due to CN orientational order). We found the scattering intensity at this position to be temperature-independent above the magnetic ordering temperature, and strongly temperature-dependent below. Our fitting process also allowed us to extract the temperature-dependent changes in magnetic Bragg peak widths, which are characteristic of the length scale of long-range magnetic order. We found that this length scale increases quickly (i.e., peaks to sharpen quickly) on cooling below the Néel temperature. The simulated PND patterns shown in Figure S11 were calculated with GSAS-II software and represent idealized magnetic crystal structure with standard time-of-flight peak-shape parameters. Note that the experimental peak shapes are much broader (especially in the case of the magnetic Bragg reflections), which is why the magnetic Bragg contribution is more easily resolved than in our experimental measurements.

### Computational Modeling

Materials modeling was performed using the Vienna ab initio simulation package (VASP version 6.2.1).^46−49^ All the electronic structure calculations were performed using density functional theory (DFT) with the nonlocal hybrid Heyd-Scuseria-Ernzerhof (HSE06) and the Perdew–Burke–Ernzerhof (PBE0) functionals. Projector-augmented wave pseudopotentials with 12, 4, and 5 valence electrons were employed for Cr, C and N, respectively. The plane wave cutoff energy was set at 500 eV and the criteria for electronic and ionic convergence were 10^–5^ eV and 0.03 eV/Å, respectively. Simulations used a Γ-centered 6 × 6 × 6 *k*-point grid. Full geometric relaxation with unconstrained total magnetic moments was performed on all modeled materials.

The unit cell and the atomic positions of Cr*^III^*[Cr^*III*^] have been optimized leading to cell parameters *a* = 10.35 and 10.41 Å, in HSE06 and PBE0, respectively, in agreement with the experimental value of *a* = 10.42 Å. It confirms that hybrid functionals allow to properly describe the atomic arrangement of the compound. In terms of magnetic properties, two magnetic orders have been considered to estimate the magnetic exchange parameter (*J*) between Cr_C_ and Cr_N_, i.e., ferromagnetic (FM) and antiferromagnetic (AFM). The calculated magnetic moments for Cr_C_ and Cr_N_ are 2.77 and −2.79 μB, respectively in HSE06 (2.78 and −2.78 μB in PBE0). These values are in excellent agreement with the experimental magnetic moment (2.78 ± 0.22 μB) deduced from PND. The tiny difference between Cr_N_ and Cr_C_ is also visible on their respective projected densities of states of the AFM order deduced from HSE06 calculations (Figure S10), which are not equivalent due to the different first neighbors for the two Cr atoms of 6 N and 6 C, respectively. As a consequence, the total density of states TDOS(up) and TDOS(down) are not the same, although the net magnetic moment in the unit cell is zero, confirming the AFM ordering. We also employed the PBE0 hybrid functional and the main features of the density of states (DOS) remain the same.

The estimation of *J* was done using the following Heisenberg Hamiltonian
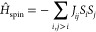
4

where *S_i_* and *S_j_* refer to the total spin vectors on atoms *i* and *j*, and the summation is over all relevant pairs (*ij*). It leads to two energy expressions (calculations carried out in the primitive cell)
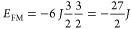
5
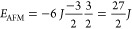
6The magnetic exchange is thus given by
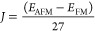
7The resulting *J*/*k*_B_ values are −100 K and −99 K in HSE06 and PBE0, respectively. The Curie–Weiss temperature (θ_CW_) can thus be estimated using the following mean-field expression:
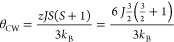
8

where *z* is the number of neighbors with which a single atom interacts with the spin exchange *J*. In the present case *z* = 6 because Cr_C_ is surrounded by 6 Cr_N_. It leads to θ_CW_ of −752 and −743 K in HSE06 and PBE0, respectively.

To estimate the Néel temperature and the evolution of the magnetic properties with temperature, we have performed Monte Carlo simulations (CMC) implemented in the Applications and Libraries for Physics Simulations code.^50^ We used the obtained *J* values from DFT calculations and a *S* = 3/2 Heisenberg model. The simulations were performed with a cell containing 1728 magnetic sites, 10,384,000 steps for the thermalization, and 90,000 Monte Carlo steps per atom for the thermodynamic averages.

We perform the CMC calculations using *J*/*k*_B_ = −100 K, to estimate the resulting magnetic susceptibility and heat capacity (Figure S11). While the transition temperature is difficult to define accurately from the magnetic susceptibility, it is simple from the heat capacity which presents a sharp transition at 323 K. Figure S12 shows the inverse or reciprocal magnetic susceptibility (χ^–1^) as a function of the temperature deduced from the CMC calculations. The Curie–Weiss fitting of the high-temperature part (from 400 to 800 K) leads to a computational prediction of the Weiss constant of −718 K.

## Supporting Information Available

Additional discussion of synthetic conditions and additional characterization (PDF)

## References

[ref1] Sato O.; Iyoda T.; Fujishima A.; Hashimoto K. Photoinduced Magnetization of a Cobalt-Iron Cyanide. Science 1996, 272, 704–705. 10.1126/science.272.5262.704. 8662564

[ref2] Ohkoshi S. I.; Arai K. I.; Sato Y.; Hashimoto K. Humidity-induced magnetization and magnetic pole inversion in a cyano-bridged metal assembly. Nat. Mater. 2004, 3, 857–861. 10.1038/nmat1260. 15558035

[ref3] Tokoro H.; Hashimoto K.; Ohkoshi S.-i. Photo-induced charge-transfer phase transition of rubidium manganese hexacyanoferrate in ferromagnetic and paramagnetic states. J. Magn. Magn. Mater. 2007, 310, 1422–1428. 10.1016/j.jmmm.2006.10.429.

[ref4] Holmes S. M.; Girolami G. S. Sol–Gel Synthesis of KV II [Cr III (CN) 6 ]·2H 2 O: A Crystalline Molecule-Based Magnet with a Magnetic Ordering Temperature above 100 °C. J. Am. Chem. Soc. 1999, 121, 5593–5594. 10.1021/ja990946c.

[ref5] Néel M. L. Propriétés magnétiques des ferrites; ferrimagnétisme et antiferromagnétisme. Ann. Phys. (Paris). 1948, 12, 137–198. 10.1051/anphys/194812030137.

[ref6] Verdaguer M.; Girolami G. S. Magnetism: Molecules to Materials; Wiley-VCH Verlag GmbH & Co. KGaA: Weinheim, Germany, 2005; Vol. 5–5, pp 283–346.

[ref7] Kahn O.; Briat B. Exchange interaction in polynuclear complexes. Part 1.—Principles, model and application to the binuclear complexes of chromium(III). J. Chem. Soc., Faraday Trans. 2 1976, 72, 268–281. 10.1039/F29767200268.

[ref8] Kahn O.; Briat B. Exchange interaction in polynuclear complexes. Part 2.—Antiferromagnetic coupling in binuclear oxo-bridged iron(III) complexes. J. Chem. Soc., Faraday Trans. 2 1976, 72, 1441–1446. 10.1039/F29767201441.

[ref9] Hay P. J.; Thibeault J. C.; Hoffmann R. Orbital interactions in metal dimer complexes. J. Am. Chem. Soc. 1975, 97, 4884–4899. 10.1021/ja00850a018.

[ref10] Gadet V.; Mallah T.; Castro I.; Verdaguer M.; Veillet P. High-Tc Molecular-Based Magnets: A Ferromagnetic Bimetallic Chromium(III)-Nickel(II) Cyanide with Tc= 90 K. J. Am. Chem. Soc. 1992, 114, 9213–9214. 10.1021/ja00049a078.

[ref11] Perlepe P.; Oyarzabal I.; Mailman A.; et al. Metal-organic magnets with large coercivity and ordering temperatures up to 242°C. Science 2020, 370, 587–592. 10.1126/science.abb3861. 33122382

[ref12] Perlepe P.; Oyarzabal I.; Voigt L.; et al. From an antiferromagnetic insulator to a strongly correlated metal in square-lattice MCl2(pyrazine)2 coordination solids. Nat. Commun. 2022, 13, 5766 10.1038/s41467-022-33342-5. 36180432 PMC9525593

[ref13] Guo F.-S.; He M.; Huang G.-Z.; Giblin S. R.; Billington D.; Heinemann F. W.; Tong M.-L.; Mansikkamäki A.; Layfield R. A. Discovery of a Dysprosium Metallocene Single-Molecule Magnet with Two High-Temperature Orbach Processes. Inorg. Chem. 2022, 61, 6017–6025. 10.1021/acs.inorgchem.1c03980. 35420419 PMC9044448

[ref14] Park J. G.; Collins B. A.; Darago L. E.; Runčevski T.; Ziebel M. E.; Aubrey M. L.; Jiang H. Z. H.; Velasquez E.; Green M. A.; Goodpaster J. D.; Long J. R. Magnetic ordering through itinerant ferromagnetism in a metal-organic framework. Nat. Chem. 2021, 13, 594–598. 10.1038/s41557-021-00666-6. 33859391

[ref15] Pedersen K. S.; Perlepe P.; Aubrey M. L.; et al. Formation of the layered conductive magnet CrCl2(pyrazine)2 through redox-active coordination chemistry. Nat. Chem. 2018, 10, 1056–1061. 10.1038/s41557-018-0107-7. 30202103

[ref16] Mallah T.; Thiebaut S.; Verdaguer M.; Veillet P. High-Tc Molecular-Based Magnets: Ferrimagnetic Mixed-Valence Chromium(III)-Chromium(II) Cyanides with Tc at 240 and 190 K. Science 1993, 262, 1554–1557. 10.1126/science.262.5139.1554. 17829385

[ref17] Sato O.; Iyoda T.; Fujishima A.; Hashimoto K. Electrochemically Tunable Magnetic Phase Transition in a High- T c Chromium Cyanide Thin Film. Science 1996, 271, 49–51. 10.1126/science.271.5245.49.

[ref18] Buschmann W. E.; Paulson S. C.; Wynn C. M.; Girtu M. A.; Epstein A. J.; White H. S.; Miller J. S. Magnetic field induced reversed (Negative) magnetization for electrochemically deposited Tc = 260 K Oxidized Films of Chromium Cyanide Magnets. Adv. Mater. 1997, 9, 645–647. 10.1002/adma.19970090812.

[ref19] Buschmann W. E.; Paulson S. C.; Wynn C. M.; Girtu M. A.; Epstein A. J.; White H. S.; Miller J. S. Reversed (Negative) Magnetization for Electrochemically Deposited High-Tc Thin Films of Chromium Hexacyanide Magnets. Chem. Mater. 1998, 10, 1386–1395. 10.1021/cm970773v.

[ref20] Nelson K. J.; Daniels M. C.; Reiff W. M.; Troff S. A.; Miller J. S. [Cr III (NCMe) 6 ] 3+ a Labile Cr III Source Enabling Formation of Cr[M(CN) 6 ] (M = V, Cr, Mn, Fe) Prussian Blue-Type Magnetic Materials. Inorg. Chem. 2007, 46, 10093–10107. 10.1021/ic7008489. 17958356

[ref21] Coronado E.; Makarewicz M.; Prieto-Ruiz J. P.; Prima-García H.; Romero F. M. Magneto-Optical Properties of Electrodeposited Thin Films of the Molecule-Based Magnet Cr5.5(CN)12·11.5H2O. Adv. Mater. 2011, 23, 4323–4326. 10.1002/adma.201101513. 21830238

[ref22] Prima-Garcia H.; Coronado E.; Prieto-Ruiz J. P.; Romero F. M. Tailoring magnetic properties of electrodeposited thin films of the molecule-based magnet Cr5.5(CN)12 11.5H2O. Nanoscale Res. Lett. 2012, 7, 232 10.1186/1556-276X-7-232. 22531148 PMC3403892

[ref23] Deeth R. J. A Theoretical Rationale for the Formation, Structure and Spin State of Pentacyanochromate(II). Eur. J. Inorg. Chem. 2006, 2006, 2551–2555. 10.1002/ejic.200600137.

[ref24] Nelson K. J.; Giles I. D.; Shum W. W.; Arif A. M.; Miller J. S. The Myth of Cyanide Always Being a Strong-Field Ligand: Synthesis and Structural Characterization of HomolepticS = 2 Pentacyanochromate(II), [CrII(CN)5]3–, and Nonacyanodichromate(II), [(CN)9]5–. Angew. Chem., Int. Ed. 2005, 44, 3129–3132. 10.1002/anie.200462763. 15832394

[ref25] Hume D. N.; Kolthoff I. M. The Oxidation Potential of the Chromocyanide—Chromicyanide Couple and the Polarography of the Chromium Cyanide Complexes 1. J. Am. Chem. Soc. 1943, 65, 1897–1901. 10.1021/ja01250a030.

[ref26] Lord R. L.; Baik M. H. Why does cyanide pretend to be a weak field ligand in [Cr(CN) 5]3-?. Inorg. Chem. 2008, 47, 4413–4420. 10.1021/ic8000653. 18433096

[ref27] Hernández-Luis F.; Rodríguez-Raposo R.; Galleguillos H. R.; Morales J. W. Solubility of Sodium Halides in Aqueous Mixtures with ϵ-Increasing Cosolvents: Formamide, N -Methylformamide, and N -Methylacetamide at 298.15 K. Ind. Eng. Chem. Res. 2016, 55, 812–819. 10.1021/acs.iecr.5b04614.

[ref28] Wessells C. D.; Peddada S. V.; McDowell M. T.; Huggins R. A.; Cui Y. The Effect of Insertion Species on Nanostructured Open Framework Hexacyanoferrate Battery Electrodes. J. Electrochem. Soc. 2011, 159, A98–A103. 10.1149/2.060202jes.

[ref29] Tromp M.; Moulin J.; Reid G.; Evans J. Cr K-Edge XANES Spectroscopy: Ligand and Oxidation State Dependence — What is Oxidation State?. AIP Conf. Proc. 2007, 882, 699–701.

[ref30] N’Diaye A.; Bordage A.; Nataf L.; Baudelet F.; Rivière E.; Bleuzen A. Toward Quantitative Magnetic Information from Transition Metal K-Edge XMCD of Prussian Blue Analogs. Inorg. Chem. 2022, 61, 6326–6336. 10.1021/acs.inorgchem.2c00637. 35414167

[ref31] N’Diaye A.; Bordage A.; Nataf L.; Baudelet F.; Rivière E.; Bleuzen A. Interplay between Transition-Metal K-edge XMCD and Magnetism in Prussian Blue Analogs. ACS Omega 2022, 7, 36366–36378. 10.1021/acsomega.2c04049. 36278067 PMC9583310

[ref32] Ojwang D. O.; Grins J.; Wardecki D.; Valvo M.; Renman V.; Häggström L.; Ericsson T.; Gustafsson T.; Mahmoud A.; Hermann R. P.; Svensson G. Structure Characterization and Properties of K-Containing Copper Hexacyanoferrate. Inorg. Chem. 2016, 55, 5924–5934. 10.1021/acs.inorgchem.6b00227. 27258790

[ref33] Mizuno M.; Ohkoshi S.; Hashimoto K. Electrochemical Synthesis of High-Tc, Colored, Magnetic Thin Films Composed of Vanadium(II/III)–Chromium(II) Hexacyanochromate(III). Adv. Mater. 2000, 12, 1955–1958.

[ref34] Deng L.; Qu J.; Niu X.; Liu J.; Zhang J.; Hong Y.; Feng M.; Wang J.; Hu M.; Zeng L.; Zhang Q.; Guo L.; Zhu Y. Defect-free potassium manganese hexacyanoferrate cathode material for high-performance potassium-ion batteries. Nat. Commun. 2021, 12, 2167 10.1038/s41467-021-22499-0. 33846311 PMC8041879

[ref35] Benitez M. J.; Petracic O.; Tüysüz H.; Schüth F.; Zabel H. Fingerprinting the magnetic behavior of antiferromagnetic nanostructures using remanent magnetization curves. Phys. Rev. B - Condens. Matter Mater. Phys. 2011, 83, 1–9. 10.1103/PhysRevB.83.134424.

[ref36] Mugiraneza S.; Hallas A. M. Tutorial: a beginner’s guide to interpreting magnetic susceptibility data with the Curie-Weiss law. Commun. Phys. 2022, 5, 95 10.1038/s42005-022-00853-y.

[ref37] Néel L. Superparamagnétisme des grains très fins antiferromagnétiques. Comptes Rendus Hebd. Des Seances L Acad. Des Sci. 1961, 252, 4075–4080.

[ref38] Richardson J. T.; Milligan W. O. Magnetic Properties of Colloidal Nickelous Oxide. Phys. Rev. 1956, 102, 1289–1294. 10.1103/PhysRev.102.1289.

[ref39] Schuele W. J.; Deetscreek V. D. Appearance of a Weak Ferromagnetism in Fine Particles of Antiferromagnetic Materials. J. Appl. Phys. 1962, 33, 1136–1137. 10.1063/1.1728633.

[ref40] Nishino M.; Yoshioka Y.; Yamaguchi K. Effective exchange interactions and magnetic phase transition temperatures in Prussian blue analogs: a study by density functional theory. Chem. Phys. Lett. 1998, 297, 51–59. 10.1016/S0009-2614(98)01081-1.

[ref41] Gubkin A. F.; Proskurina E. P.; Kousaka Y.; Sherokalova E. M.; Selezneva N. V.; Miao P.; Lee S.; Zhang J.; Ishikawa Y.; Torii S.; Kamiyama T.; Campo J.; Akimitsu J.; Baranov N. V. Crystal and magnetic structures of Cr13NbSe2 from neutron diffraction. J. Appl. Phys. 2016, 119, 013903 10.1063/1.4939558.

[ref42] Rodic D.; Antic B.; Tellgren R.; Rundlof H.; Blanusa J. A change of magnetic moment of Cr ion with the magnetic phase transition in CuCr2Se4. J. Magn. Magn. Mater. 1998, 187, 88–92. 10.1016/S0304-8853(98)00106-1.

[ref43] Entley W. R.; Girolami G. S. High-Temperature Molecular Magnets Based on Cyanovanadate Building Blocks: Spontaneous Magnetization at 230 K. Science 1995, 268, 397–400. 10.1126/science.268.5209.397. 17746547

[ref44] Zheng X. G.; Xu C. N.; Nishikubo K.; Nishiyama K.; Higemoto W.; Moon W. J.; Tanaka E.; Otabe E. S. Finite-size effect on Néel temperature in antiferromagnetic nanoparticles. Phys. Rev. B 2005, 72, 014464 10.1103/PhysRevB.72.014464.

[ref45] Toby B. H.; Von Dreele R. B. GSAS-II: the genesis of a modern open-source all purpose crystallography software package. J. Appl. Crystallogr. 2013, 46, 544–549. 10.1107/S0021889813003531.

[ref46] Kresse G.; Furthmüller J.; Hafner J. Theory of the crystal structures of selenium and tellurium: The effect of generalized-gradient corrections to the local-density approximation. Phys. Rev. B 1994, 50, 13181–13185. 10.1103/PhysRevB.50.13181. 9975508

[ref47] Kresse G.; Furthmüller J. Efficiency of ab-initio total energy calculations for metals and semiconductors using a plane-wave basis set. Comput. Mater. Sci. 1996, 6, 15–50. 10.1016/0927-0256(96)00008-0. 9984901

[ref48] Perdew J. P.; Burke K.; Ernzerhof M. Generalized Gradient Approximation Made Simple. Phys. Rev. Lett. 1996, 77, 3865–3868. 10.1103/PhysRevLett.77.3865. 10062328

[ref49] Heyd J.; Scuseria G. E.; Ernzerhof M. Hybrid functionals based on a screened Coulomb potential. J. Chem. Phys. 2003, 118, 8207–8215. 10.1063/1.1564060.

[ref50] Bauer B.; Carr L. D.; Evertz H. G.; et al. The ALPS project release 2.0: open source software for strongly correlated systems. J. Stat. Mech.: Theory Exp. 2011, 2011, P05001 10.1088/1742-5468/2011/05/P05001.

