# Supplementary material for: Nonaqueous Synthesis of Low-Vacancy Chromium Hexacyanochromate
Source: Inorg Chem. 2024 Nov 18;63(48):22856–64. doi: 10.1021/acs.inorgchem.4c03856 (PMC11615938; doi:10.1021/acs.inorgchem.4c03856)
Supplement: Supplementary file 1 — ic4c03856_si_001.pdf [file ic4c03856_si_001.pdf]

Supporting Information

Non-aqueous synthesis of low-vacancy chromium hexacyanochromate

Maximilian Schart[1], Ramón Torres-Cavanillas[1], Samuel Wheeler[1], Kevin Hurlbutt[1], Pascal Manuel[2], Dmitry Khalyavin[2], Ruomu Zhang[1], David Vincent[3], Xavier Rocquefelte[3], George Volonakis[3], Andrew Goodwin[4], Lapo Bogani[1], and Mauro Pasta[1*]

[1]Department of Materials, University of Oxford, OX1 3PH, U.K.
[2]ISIS Pulsed Neutron and Muon Source, STFC Rutherford Appleton Laboratory, Harwell Campus, Didcot, Oxon, OX11 0QX, U.K.
[3]Univ Rennes, ENSCR, INSA Rennes, CNRS, ISCR (Institut des Sciences Chimiques de Rennes), UMR 6226, Rennes F-35000, France
[4]Inorganic Chemistry Laboratory, Department of Chemistry, University of Oxford, Oxford OX1 3QR, U.K.
[*]Corresponding author: mauro.pasta@materials.ox.ac.uk

Supplementary discussion

Analysis of synthesis conditions

Because of the crystal symmetry, the intensity of the (111) reflection in the materials' XRD patterns is indicative of Cr deficiencies on one sublattice, that is, vacancies. This can be seen from the computed diffractograms of idealised structures (fig. S1, bottom). An overview of the XRD patterns of the materials synthesised via all tested conditions is shown in fig. S1, top. It is evident that using FA as reaction medium and Na^+, K^+, Rb^+ as counterions yields highly vacant materials. In the same solvent, using Cs^+ leads to a low-vacancy material. Using NMF and Na^+, K^+ counterions leads to amorphous precipitate with low yield. In NMF reaction medium and Rb^+, Cs^+, low-vacancy, crystalline materials form. The materials formed with Cs^+, while low-vacancy, are not regarded as suitable for further chemical oxidation because of the difficulties in removing the large inserted ions from the interstices (see e.g. [51,52]).

Supplementary figures

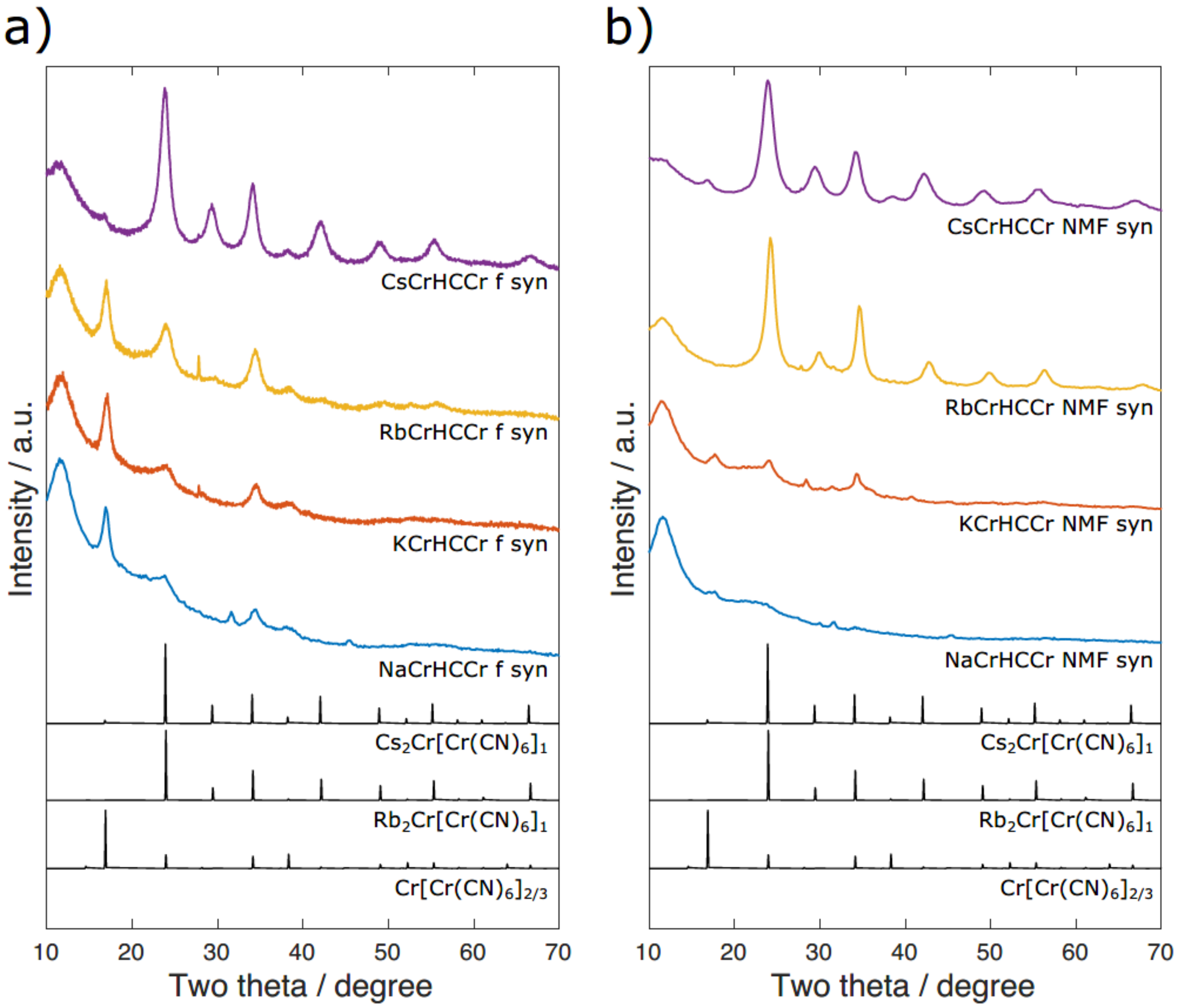

Figure S1: Overview of tested synthesis conditions. XRD patterns of materials synthesised in FA (a) and NMF (b) with different counterions (coloured, top). Simulated diffractograms of idealised, zero- and high-vacancy structures are shown for reference (black, bottom).

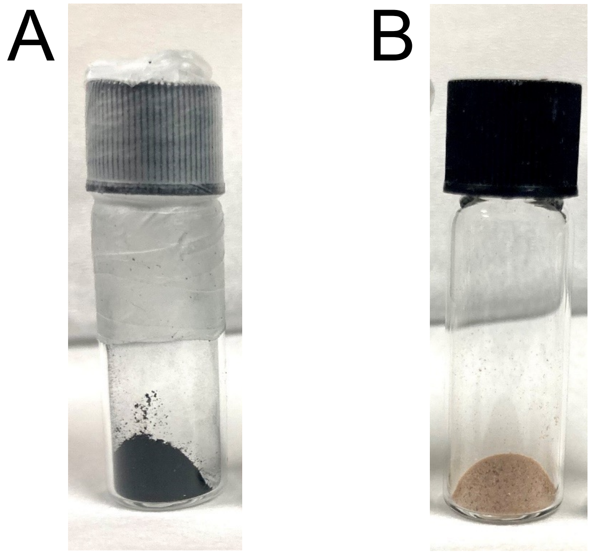

Figure S2: Photographs of (1) (A) and (2) (B).

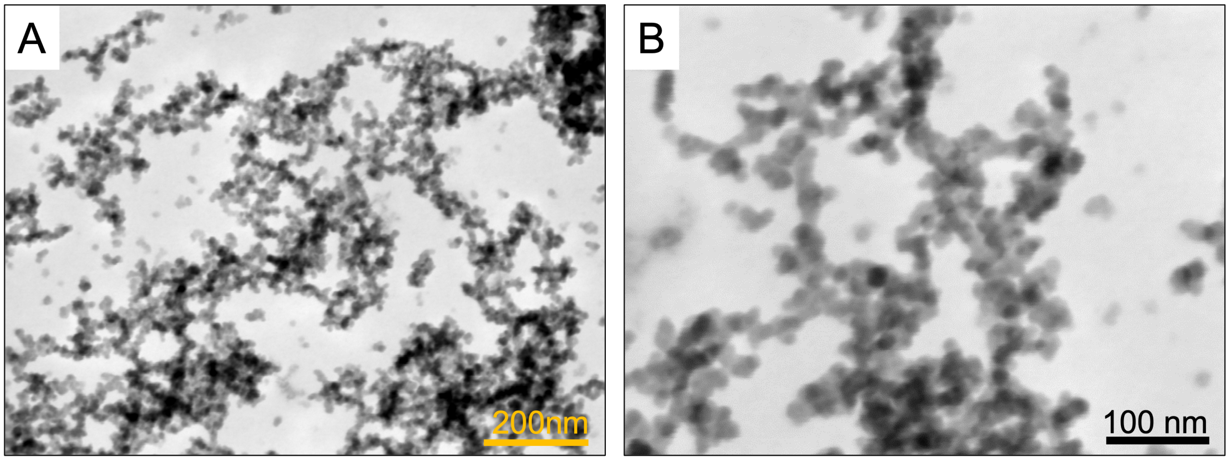

Figure S3: Transmission-mode SEM of material (1) at different magnifications. Low magnification (A), high magnification (B).

Sample	Rb / µg l^{-1}	Cr / µg l^{-1}	Rb:Cr at%	Deviation
(1)	22.0	18.4	0.727	0.010
	22.3	18.6	0.729	0.020
	23.5	18.7	0.764	0.003
	34.1	28.4	0.730	0.012
Average			0.738	0.013
(2)	-0.0762	30.53	-0.00152	0.00053
	-0.532	26.4	-0.00631	0.00045
	-0.274	35.3	-0.00282	0.00015
	-0.164	40.3	-0.000672	0.000345
Average			-0.00283	0.00039

Figure S4: ICP-MS results of three individual digestions of materials (1) and (2).

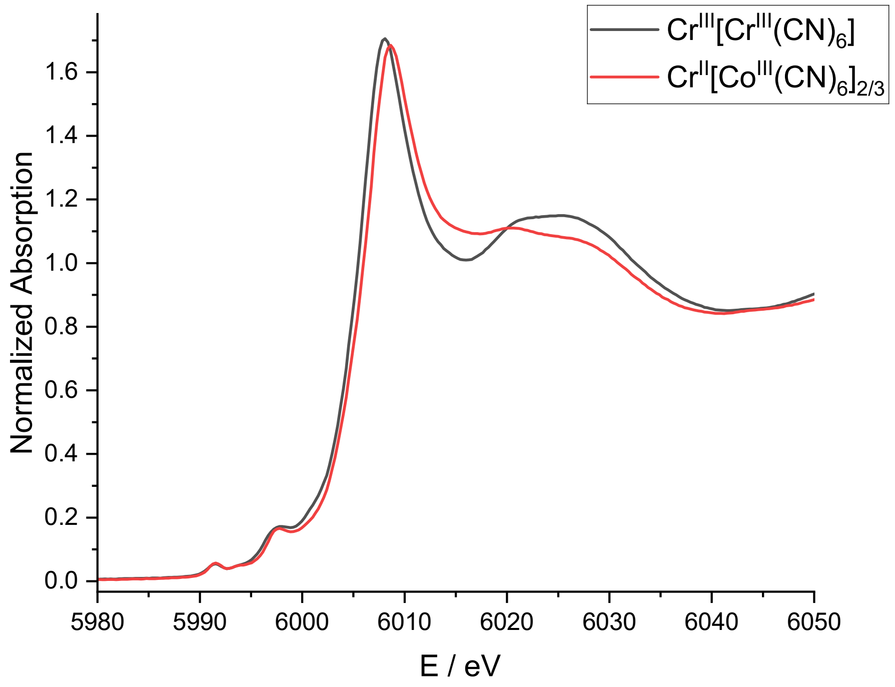

Figure S5: Comparison of material (2) and $Cr^{II}[Co^{III}(CN)_6]$ spectra at the Cr K-edge XANES over the 5980–6050 eV energy range.

Atom	Multiplicity	Occupancy	x	y	z	Uiso
Cr(1)	4	0.997(19)	0	0	0	0.021(2)
Cr(2)	4	1	0.5	0	0	0.021(2)
C	24	0.997(19)	0.200(2)	0	0	0.024(3)
N	24	0.997(19)	0.301(2)	0	0	0.024(3)
Rb	8	0.839(13)	0.25	0.25	0.25	0.079(2)
Global: crystallite size: 17 nm, lattice parameter a = 10.3142(5) Å, R=2.99%, wR=4.29%, χ^2=30419.1, GOF=2.30						

Figure S6: Results of Rietveld refinement of material (1).

Atom	Multiplicity	Occupancy	x	y	z	Uiso
Cr(1)	4	0.996(7)	0	0	0	0.0111(4)
Cr(2)	4	1	0.5	0	0	0.0111(4)
C	24	0.996(7)	0.175(1)	0	0	-0.00001(117)
N	24	0.996(7)	0.2931(4)	0	0	-0.00001(117)
Global: crystallite size: 13 nm, lattice parameter a = 10.43542 Å, R=2.17%, wR=3.05%, χ^2=105099, GOF=3.63						

Figure S7: Results of Rietveld refinement of material (2).

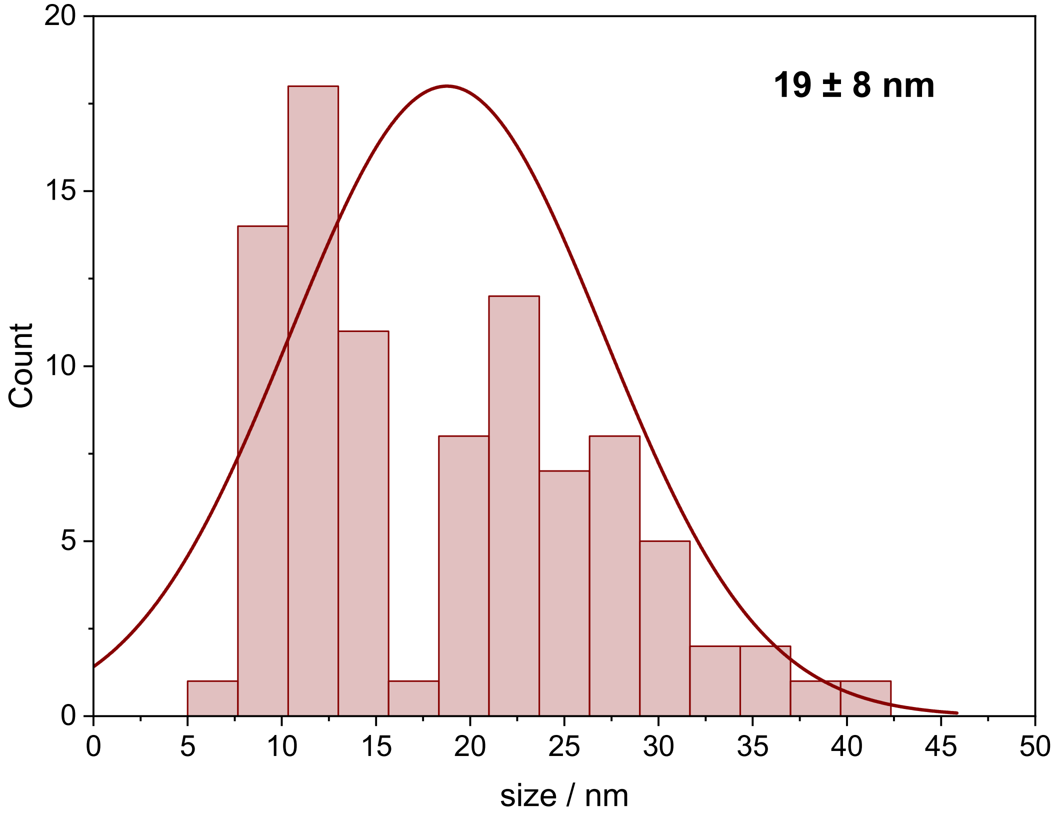

Figure S8: Particle size distribution of material (2).

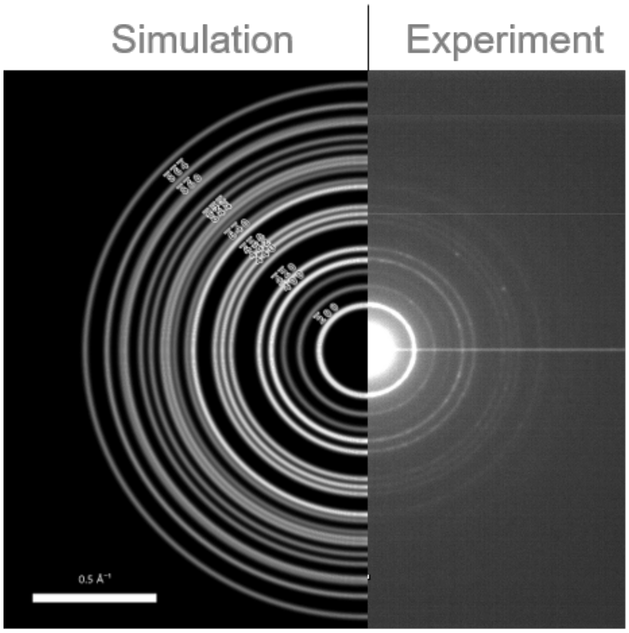

Figure S9: Selected area electron diffraction of material (2).

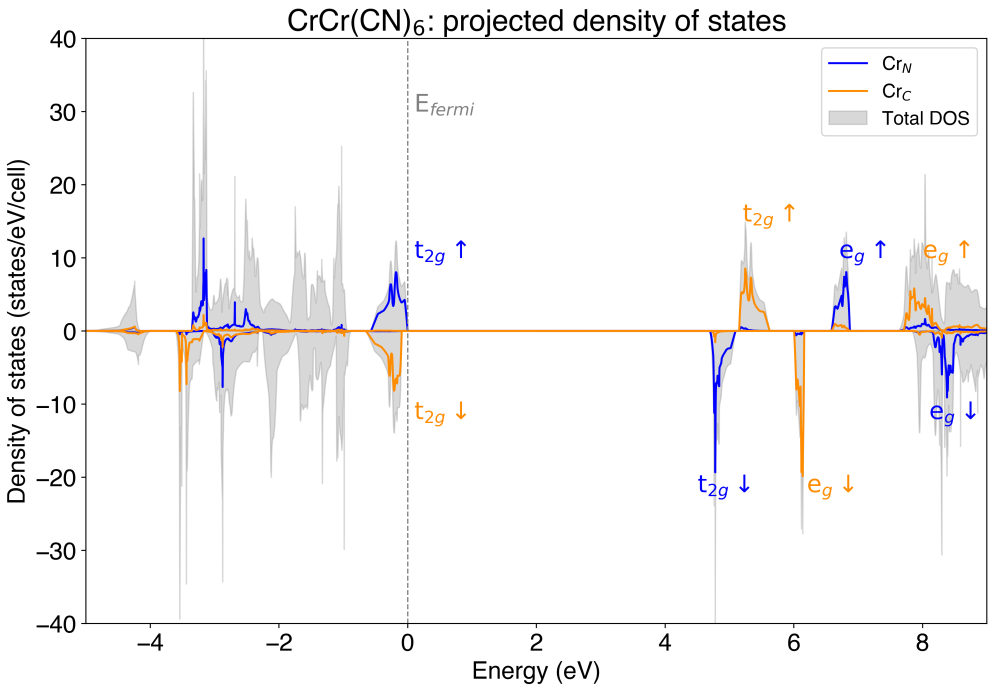

Figure S10: Total and projected density (pDOS) of states of $CrCr(CN)_6$ with an AFM order obtained from HSE06 hybrid DFT calculations. The pDOS of Cr_C and Cr_N are depicted in orange and blue respectively. The Fermi level has been defined as the energy reference at 0 eV.

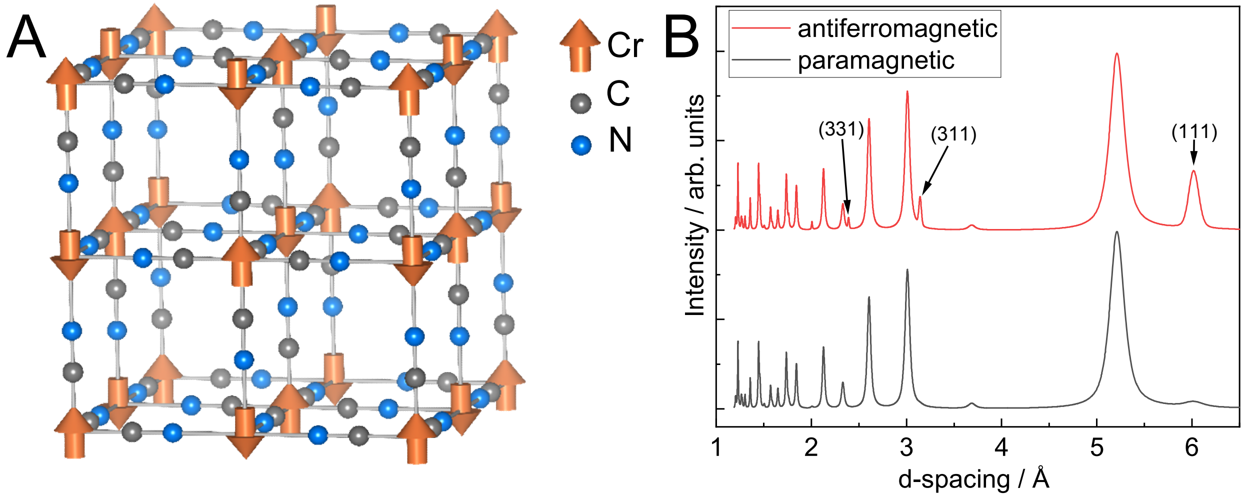

Figure S11: Spin arrangement (A) and simulated PND patterns (B).

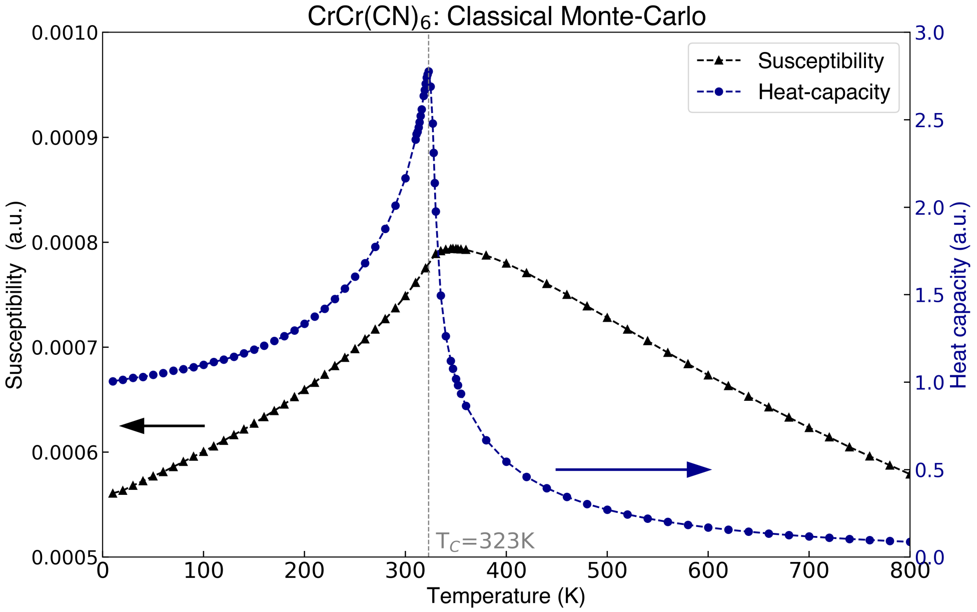

Figure S12: Magnetic susceptibility and heat capacity deduced from classical Monte-Carlo simulations using J/k_B = -100 K.

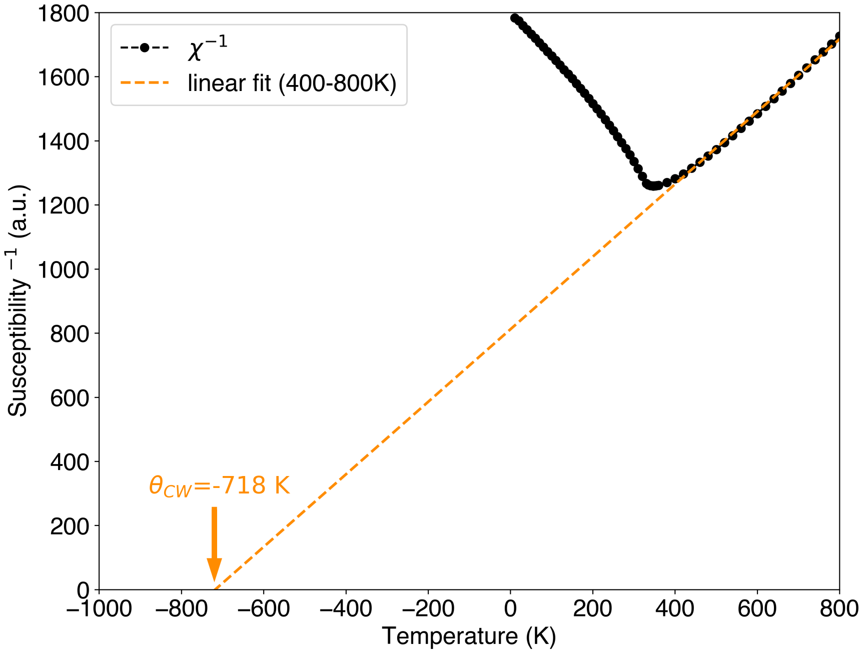

Figure S13: Inverse of the magnetic susceptibility (χ^{-1}) from classical Monte-Carlo simulations using J/k_B = -100 K. The ϑ_{CW} is calculated by a linear fit at the hight temperature range between 400-800 K.

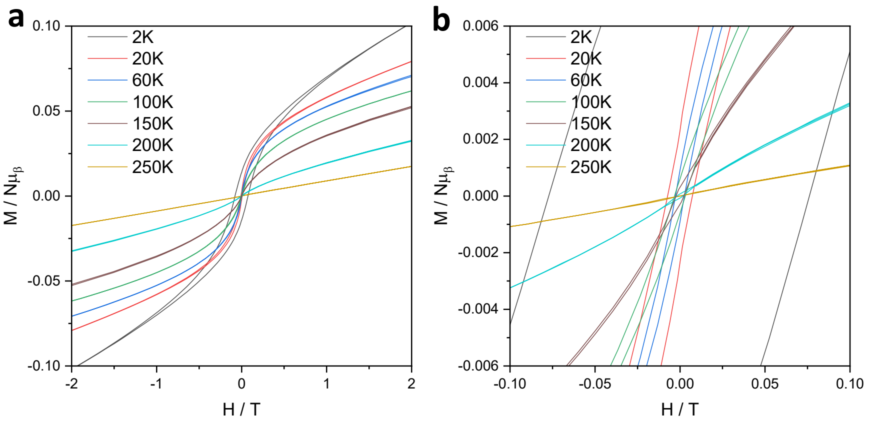

Figure S14: (a) Magnetization against the applied field for material (2) at different temperatures and a zoom between 0.1 and -0.1 T and (b) Field-dependent magnetisation measurements of material (2) from 2 K to 250 K

.

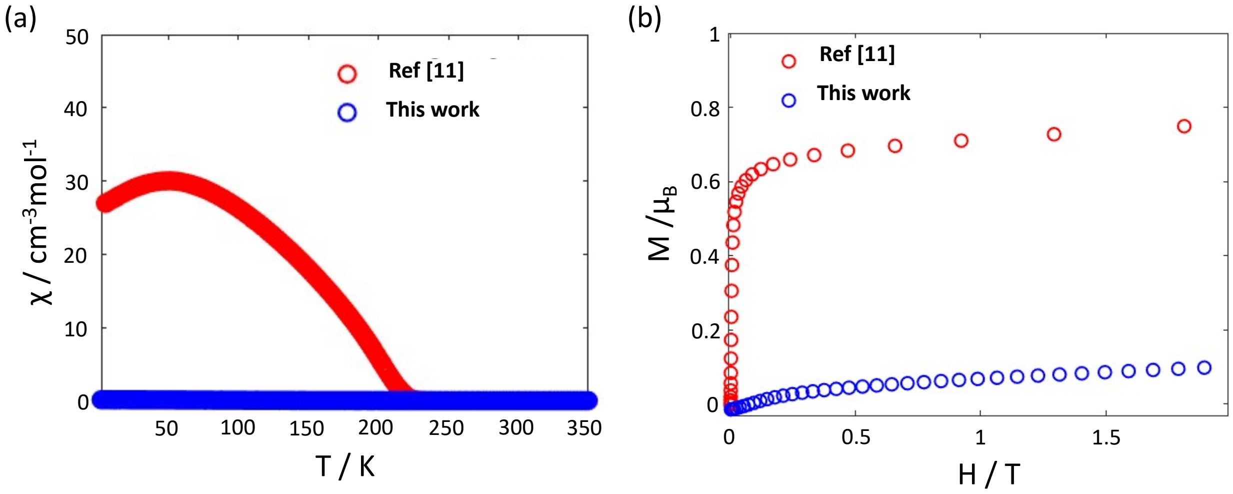

Figure S15: a) Comparison of the thermal variation of the χ vs T for material (2), in blue, and following the same protocol as in reference 11, in red. b) Magnetization against the applied field of the same compounds

.

References

[51] Yutaka Moritomo et al. "Size Dependent Cation Channel in Nanoporous Prussian Blue Lattice". *Appl. Phys. Express* 2.8 (2009), p. 085001.
[52] Akira Takahashi et al. "Unveiling Cs-adsorption mechanism of Prussian blue analogs: Cs^{+}-percolation via vacancies to complete dehydrated state". *RSC Adv.* 8.61 (2018), pp. 34808–34816.
